# 22^nd^ AMN Congress in Bangkok, Thailand – Interview with Prof. Stefanie Duchac

**DOI:** 10.25122/jml-2025-1007

**Published:** 2025-10

**Authors:** Stefana-Andrada Dobran, Alexandra Gherman

**Affiliations:** 1 RoNeuro Institute for Neurological Research and Diagnostic, Cluj-Napoca, Romania; 2 Sociology Department, Babes-Bolyai University, Cluj-Napoca, Romania

**Interviewee: Professor Stephanie Duchac**

**Interviewer: Stefana-Andrada Dobran**

During the 22^nd^ Congress of the Academy for Multidisciplinary Neurotraumatology (AMN), which has taken place in Bangkok, Thailand, on July 4-5, Prof. Stefanie Duchac offered her perspective on managing post-traumatic brain injury (TBI) dysphagia. Moreover, she was part of the faculty at NTSC Extended AMN Intensives course on 3^rd^ of July, where she shared insight on collaborative approaches and rehabilitation aspects of dysphagia post-TBI.

Prof. Duchac is a professor of speech and language therapy at the SRH University in Germany, with extensive clinical experience in diagnosing and treating patients with dysphagia following stroke and TBI. Academically, she participates in several clinical research projects and is an active board member of the European Society of Swallowing Disorders (ESSD), where she now heads the ESSD Academy.

She supports interprofessional dysphagia teams and frequently conducts seminars and workshops, primarily on evidence-based dysphagia management and videofluoroscopy of swallowing. Prof. Duchac is the co-founder of the first German-language dysphagia podcast "IssNix!", the initiator of a national VFSS-register and the creator of a dysphagia mentorship program. As keynote speaker (e.g. TEDx) she works to raise public awareness for dysphagia.


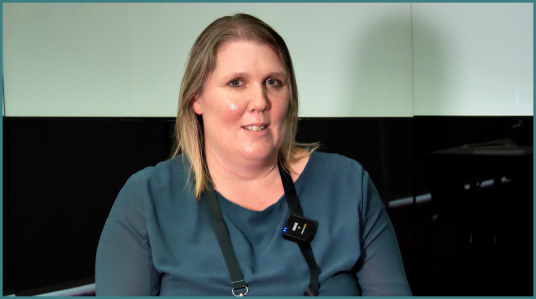


She is also an active member of the European Federation of NeuroRehabilitation (EFNR), participating as a trainer at the EFNR Dysphagia Course Series in 2023 in Uzbekistan and at the EFNR Rehabilitation Course Series in Poland this year.


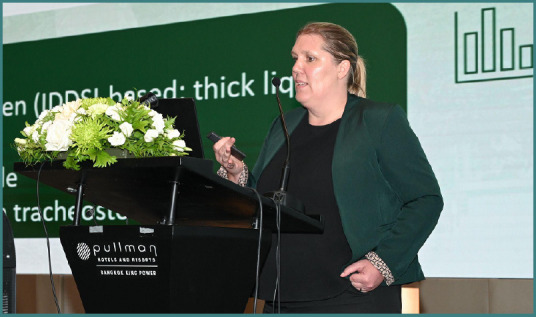


**S.D.: Dear professor Stephanie Duchac, welcome to the 22^nd^ Congress of the AMN here in Bangkok, Thailand. What is your perspective of the AMN as a global player in the practice and science fields of TBI?**

Stefanie Duchac: First of all, thank you very much for having me as an invited speaker and giving me the opportunity to experience this congress, which is really amazing. In my view, AMN really plays a major role when it comes to TBI care, and it plays a unique and also very much needed, role in the global perspective. Given what Prof. Muresanu has mentioned this morning in his mission and vision, the different aspects that AMN tries to achieve when it comes to education, clinical practice, but also research and advocacy, I really think that AMN is perfect in connecting all these different parts. And especially in my view as an SLP – speech and language pathologist –, I really like the multidisciplinary perspective of the whole society. So, I really think it is a major aspect when it comes to a very holistic approach in patient care and patient-centered care.

**S.D: The 2025 Congress was preceded by the first edition of the NTSC Extended - AMN Intensives teaching course in Thailand. As a faculty member, please share with us your opinion regarding this educational event**.

Stefanie Duchac: This event was truly inspiring for me, and this was mainly with two aspects. On the one hand, from my side as an SLP, to really dive into the world of the neurosurgery of traumatic brain injury with all the hands-on courses and to be part of this really practical guided course was truly amazing, and I learned a lot. On the other side, I was amazed of the interest of the participants when it came to a topic such as swallowing disorders and dysphagia, which is not on their daily routine, but they are very interested in acknowledging that TBI is not just acute care, but also there is some path following it, and rehabilitation is a major part of it, especially in my perspective, the swallowing rehabilitation, of course, and the communication of the patients. That was really a unique experience, and I appreciated that I could be part of this.


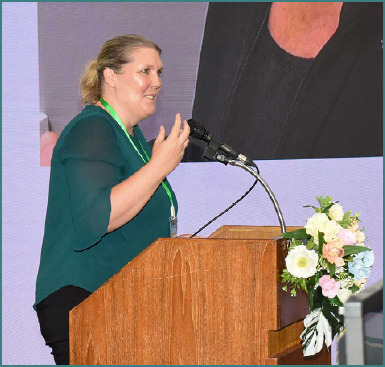


**S.D.: As a professor of speech and language therapy, what is your intake related to the 2025 AMN congress, and how do you foresee future developments in the complex field of multidisciplinary TBI?**

Stefanie Duchac: As an SLP, the multidisciplinary approach is a huge impact for me, and I'm honored to be here and represent the profession of speech and language pathologies and raise awareness for complications such as dysphagia, but also communication problems that are a major complication following TBI. So, for the future, I am positive that systematically including other healthcare professionals and giving space to the whole rehab time of a TBI patient, I think might be a really important step for the whole rehabilitation process of these patients.


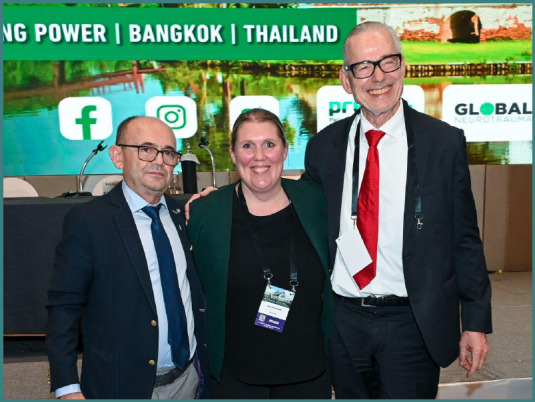


**S.D.: At a personal level, how would you envision your future collaboration within the AMN academic environment?**

Stefanie Duchac: For me, AMN represents a unique and highly valuable platform where interprofessional exchange, networking, research, and clinical practice come together for the benefit of our patients. Looking ahead, I envision my future collaboration within AMN in several ways: by actively sharing my expertise, supporting colleagues and younger professionals in the field, and at the same time continuing to learn from the diverse perspectives represented in this inspiring community. I strongly believe that through this joint effort we can not only broaden our scientific knowledge but also directly contribute to improving patient care worldwide. To be part of such a mission is both an honor and a great joy for me, and I am highly motivated to remain an active member of AMN in the years to come.

